# *Treponema* *pallidum* Dysregulates Monocytes and Promotes the Expression of IL-1β and Migration in Monocytes Through the mTOR Signaling Pathway

**DOI:** 10.3389/fcimb.2020.592864

**Published:** 2020-11-13

**Authors:** Wen-Na Liu, Xiao-Yong Jiang, Yan-Zhu Xu, Xiao-Han Sun, Kai-Xuan Wu, Xin-Lin Hu, Yong Lin, Li-Rong Lin, Man-Li Tong, Li-Li Liu

**Affiliations:** ^1^ Center of Clinical Laboratory, Zhongshan Hospital, School of Medical, Xiamen University, Xiamen, China; ^2^ Department of Laboratory Medicine, Sichuan Provincial People’s Hospital, University of Electronic Science and Technology of China, Chengdu, China; ^3^ Department of Dermatology, Zhongshan Hospital, School of Medical, Xiamen University, Xiamen, China

**Keywords:** mTOR, IL-1β, migration, monocytes, *Treponema pallidum*

## Abstract

Monocytes are widely involved in the body’s defense response, and abnormally regulated monocyte subsets are closely related to the pathogenesis of various diseases. It is unclear whether *Treponema pallidum* (*Tp*) dysregulates monocyte subsets and impacts the functions of monocytes. This study aims to analyze the distribution of monocyte subsets in syphilis patients and the effect of *Tp* on monocyte functions to explore the pathogenesis of syphilis. Flow cytometry was employed to detect monocyte subsets. With or without pre-treatment with rapamycin, THP-1 cell migration stimulated by *Tp* was investigated by a Transwell migration assay, and THP-1 cell phagocytosis was studied using fluorescent microspheres. IL-1β and TNF-α expression was quantified by PCR and flow cytometry, while LC3 and mTOR were investigated in *Tp*-exposed THP-1 cells using western blotting. *Tp* infection led to an increase in the proportion of CD14^++^CD16^+^ monocytes and a decrease in the proportion of CD14^++^CD16^-^ monocytes. In addition, *Tp* promoted monocyte (THP-1) CD14 and CD16 expression *in vitro*, induced the expression of IL-1β and TNF-α in a dose-dependent manner and promoted the migration and autophagy of monocytes. Furthermore, mTOR phosphorylation on monocytes was stimulated by *Tp*, and the levels peaked at 30 min. Pre-treatment with rapamycin (mTOR inhibitor) attenuated the expression of IL-1β and migration in *Tp*-exposed THP-1 cells. *Tp* abnormally regulates monocyte subsets and promotes migration, autophagy, and the expression of IL-1β and TNF-α in THP-1 cells. Meanwhile, the mTOR affected the expression of IL-1β and migration in *Tp*-exposed THP-1 cells. This study is important as it sheds light on the mechanism by which monocytes interact with *Tp* during infection.

## Introduction

Syphilis caused by *Treponema pallidum* (*Tp*) continues to be a prevalent disease, which raises public health concerns. The number of new syphilis cases reported in China increased to 587,464 in 2019 (https://www.cdc.gov/). Although the advent of penicillin is crucial to the treatment of syphilis, syphilis remains a worldwide public health problem. *Tp* infection triggers a complex host response that involves both innate and adaptive immunity (Rekart et al., 2017). Recently, increasing evidence has shown that host innate immune responses play critical roles in the containment of *Tp* infection (Salazar et al., 2007).

Monocytes are essential components of the innate immune system, accounting for approximately 10% of circulating leukocytes in humans and are responsible for phagocytosis of pathogens and dead cells, as well as antitumor activities (Parihar et al., 2010). A recent study suggests that circulating monocytes are important participants in the defense against a wide range of microbial pathogens, and monocyte polarization may differ in the development of different diseases (Serbina et al., 2008; Andrade et al., 2014). Based on the expression patterns of Fc*γ*RIII, also known as CD16, and the LPS receptor CD14, circulating human monocytes are classified into three subsets: classical monocytes (CD14^++^CD16^−^ monocytes), non-classical monocytes (CD14^+^CD16^++^ monocytes) and intermediate monocytes (CD14^++^CD16^+^ monocytes). These different monocyte subpopulations can exhibit some distinct functional roles in a range of homeostatic and pathological conditions. It was reported that the heterogeneous monocyte population exerts its immune function through migration, phagocytosis, secretion of cytokines (Kapellos et al., 2019). In healthy donors, classical monocytes (CD14^++^CD16^−^ monocytes) were primed for phagocytosis, innate sensing/immune responses and migration using a novel single-cell PCR gene expression analysis tool (Gren et al., 2015); however, *Brucella* infection led to an increase in the proportion of classical monocytes, which showed downregulation of immune responses (Wang Y. et al., 2017). Meanwhile, Nabatanzi et al. (2019) observed that the IL-1β production of classical monocytes among human immunodeficiency virus (HIV)-positive patients who received antiretroviral therapy was lower than that of those among healthy HIV-negative adults. Some studies have found that intermediate monocytes were well-suited for antigen presentation and cytokine secretion, while Szaflarska and colleagues (Szaflarska et al., 2004) described an antitumoral phenotype of these cells. Zawada et al. (2016) discovered that the distinct functional properties of monocyte subsets rely on the differential methylation status of immune-related genes. However, the exact role of monocyte subsets in immunity remains elusive, as there are different viewpoints toward this matter. To summarize, each monocyte subset has different features in different homeostasis and disease states. To the best of our knowledge, no detailed analysis of the different monocyte subsets in *Tp* infection and the effects of *Tp* on monocyte function has been reported up to date.

The goal of the present study was to analyze the peripheral blood mononuclear cell subset distribution of syphilis patients, meanwhile, to explore the pathogenic effects of *Tp* on monocyte polarization, inflammatory cytokine expression and migration *in vitro.*

## Methods

### Study Participants

This study was conducted between March 2019 and October 2019 at Zhongshan Hospital, School of Medicine, Xiamen University, China. During the study period, 55 syphilis patients diagnosed at Zhongshan Hospital, Xiamen University, were included in the study, and the same number of healthy controls were recruited. The selection criteria adhered to the United States Centers for Disease Control and Prevention guidelines (Workowski and Bolan, 2015), the European Centre for Disease Prevention and Control guidelines, and our previous report (Janier et al., 2014). Specimens from all participants were collected as previously published (Liu et al., 2019); whole blood was collected in 2-ml tube that contained EDTA-K2 for the flow cytometry assays, and subjects who had co-infection with human immunodeficiency virus (HIV), hepatitis B virus (HBV), and hepatitis C virus (HCV) were excluded. This study was approved by the Ethics Committee of Zhongshan Hospital, Xiamen University and was in accordance with the Declaration of Helsinki.

### Flow Cytometry Assays

Flow cytometry was performed on a Mindray BriCyte E6 flow cytometer (Mindray, Shenzhen, China) as described in our previous paper (Liu et al., 2019). Briefly, participants’ peripheral blood samples and monocytes (THP-1 cells) were stained with the following antibodies: mouse anti-human CD14-PE and mouse anti-human CD16-PE-Cy7, anti-human gamma 1-PE and anti-human gamma 1-PE-Cy7 for non-fluorescent and non-specific fluorescence controls. *In vitro* experiments, intracellular staining was performed using the following mouse anti-human mAbs: TNF-α-PE-Cy7 and IL-1β-FITC. Antibodies were purchased from BioLegend (San Diego, CA, USA), except the anti-CD14-PE antibody (BD Biosciences, San Jose, CA, USA). Data were analyzed with FlowJo (TreeStar Software, Ashland, OR, USA).

### Cell Culture

THP-1 cells (purchased from the American Type Culture Collection, Manassas, VA, USA) were incubated in RPMI-1640 medium (HyClone, Logan, USA) supplemented with 10% heat-inactivated fetal bovine serum (Biological Industries Ltd., Kibbutz Beit HaEmek, Israel), 100 U/ml penicillin, and 100 g/ml streptomycin (Invitrogen/Life Technologies, Carlsbad, CA, USA) at 37°C in 5% CO_2_. For stimulation experiments, THP-1 cells incubated were with *Tp* at different multiplicities of infection (MOIs) (*Tp*: cells 1:1, 10:1 and 20:1) at 37°C in 5% CO_2_ for 12 h in 12-well culture plates. Concurrently, the autophagy and mTOR were assessed by adding the inhibitors, which are 3-methyladenine (3-MA) (1 mM) and rapamycin (Rapa) (50 nM), respectively, dissolved in PBS to pre-treat the cells for 30 min. 3-MA and Rapa were purchased from Sigma-Aldrich (St. Louis, MO, USA).

### Propagation of *Tp*

The Nichols strain was kindly donated by Lorenzo Giacani, PhD (University of Washington, Seattle, USA) and was maintained as previously described (Gao et al., 2018). The strain was propagated intratesticularly in adult male New Zealand white rabbits.

### Real-Time PCR Assay

In order to evaluate the mRNA expression, total RNA was extracted from the cultured cells using an RNeasy kit (Tiangen Biotech Co., Ltd., Beijing, China) and then reverse transcripted with a high-capacity cDNA reverse transcription kit (Takara, Shiga, Japan) and sequenced using the Illumina HiSeq2500 platform by Gene Denovo Biotechnology Co (Guangzhou, China). DESeq2 software was performed. RNA differential expression analysis and principal component analysis were performed with R package gmodels (http://www.rproject.org/). Gene Ontology (GO) enrichment analysis provides all GO terms that are significantly enriched in differentially expressed genes (DEGs) comparing to the genome background, and filter the DEGs that correspond to biological functions. RT-PCR was carried out using a QuantiFast SYBR One-Step RT-PCR kit (Qiagen, Shanghai, China) and the LightCycler R96 instrument (Roche Diagnostics, Roche Instrument Center AG, Rotkreuz, Switzerland). The expression level was normalized with reference to the GAPDH housekeeping gene, and the relative gene expression was calculated using the 2^−ΔΔCT^ method. The primers are listed in **Supplementary Table 1**.

### Western Blotting Assays

Western blotting analysis was performed according to the method described in the literature (Deng et al., 2016). The primary (rabbit anti-human LC3, rabbit anti-human mTOR and mouse anti-human GAPDH antibodies) and secondary (anti-rabbit horseradish peroxidase-labeled antibody and anti-mouse horseradish peroxidase-labeled antibody) antibodies were purchased from Cell Signaling Technology (Danvers, MA, USA) and used at a dilution of 1:1,000 and 1:10,000, respectively. The results were quantified using ImageJ software.

### Cell Migration Assays

For cell migration experiments, cells cocultured with *Tp* (MOI = 10) for 12 h were collected in 24-well Transwell plates (Corning Incorporation, NY, USA) Approximately 5 × 10^5^ cells in a volume of 200 µl (serum free) were seeded in the upper chamber, and then 400 µl of RPMI 1640 medium with 10% FBS was added to the lower chambers of Transwell plates. The Transwell plates were incubated at 37°C with 5% CO_2_ for 2, 4, and 6 h. Cells that migrated to the lower chamber were counted by flow cytometry, in which flow cytometry absorbed 30 µl of cell suspension and calculated the cell concentration of each group.

### Phagocytosis Assays

Phagocytic capacity was assayed by flow cytometry using FITC-dextran. *Tp*-treated cells, and an equal amount of PBS-treated cells as a control group was collected, and the removed cells (5 × 10^5^) suspended in RPMI 1640 medium with 10% FBS were incubated together with FITC-dextran (0.1 mg/ml) at 37°C for 2, 4, and 6 h as described previously (Aryanpur et al., 2016). The cells were washed with PBS three times, and flow cytometry was used to detect the phagocytic rate of each group of cells.

### Statistical Analysis

SPSS version 19.0 (SPSS Inc., Chicago, IL, USA) was used for statistical analysis of the experimental data. To be more specific, Shapiro–Wilk test was used to examine the normal distribution of continuous variables. Two-tailed Student’s *t* test and one-way analysis of variance (ANOVA) were used to compare means. A 2-sided *P* value less than 0.05 was considered statistically significant.

## Results

### Monocyte Subsets Associated With Syphilis

**Figure 1:**
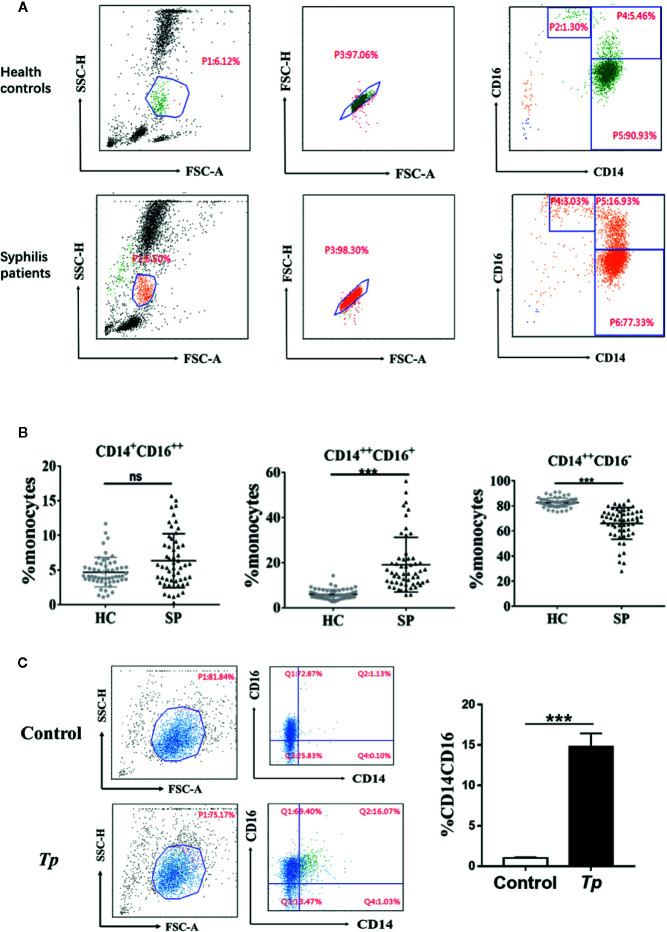
Characteristics of monocyte subsets in syphilis patients and healthy controls. **(A)** Gating strategy for monocyte subsets. **(B)** The percentage of each monocyte subset in syphilis patients (n = 55) and healthy controls (n = 55). **(C)** *Tp* promoted the expression of CD14 and CD16 in THP-1 cells. The data in the bar graphs are the means ± SEMs of three separate experiments. ***P < 0.001. FSC, forward scatter; SSC, side scatter; HC, healthy controls; SP, syphilis patient.

Among the 55 syphilis patients, 32 were male, and 23 were female. The median age of syphilis patients was 56 years (range: 38 to 74). All syphilis patients were subjected to reactive baseline serum RPR and serum TPPA tests. Among the 55 subjects in the control group, 23 were male, and 32 were female. The median age of the participants was 52 years (range: 39 to 65), and serum RPR and serum TPPA tests were negative. The characteristics of the study participants are summarized in **Supplementary Table 2**. To determine whether *Tp* affected the frequency and phenotype of monocyte subsets, cells were analyzed by flow cytometry, and the gating strategy is shown in **Figure 1A**. Based on CD14 and CD16 expression, the monocytes were divided into three subsets. The proportion of the intermediate monocyte (CD14^++^CD16^+^ monocytes) subsets was significantly elevated in syphilis patients compared to healthy controls (15.06 ± 10.75 *vs.* 5.52 ± 2.89%, *P* < 0.001), whereas the proportion of classical monocytes (CD14^++^CD16^–^ monocytes) was significantly lower in syphilis patients than in healthy controls (68.97 ± 14.45% *vs.* 82.52 ± 4.56%, *P* < 0.001) (**Figure 1B**).

The effect of *Tp* on monocytes was further examined *in vitro*. When *Tp* was co-cultured with THP-1 cells, the proportion of the CD14^+^CD16^+^ monocyte subset significantly increased, compared with that of the control group (14.81 ± 0.9% vs. 1.03 ± 0.05%, *P* < 0.001, **Figure 1C**).

### Differential Expression of mRNAs in Cells Between *Tp*-Exposed Monocytes THP-1 and Control Groups

**Figure 2:**
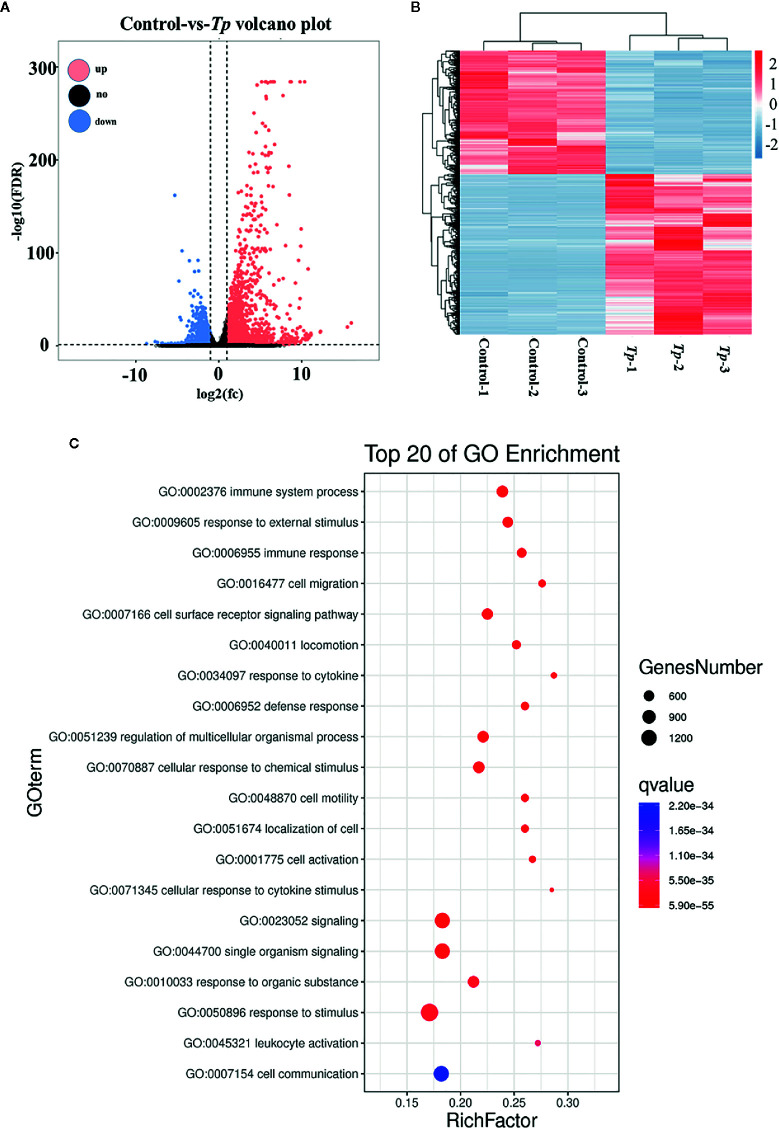
Differential expression of mRNAs in cells between the *Tp* and control groups. The volcano plots **(A)** and heatmap **(B)** demonstrate the differentially expressed mRNAs between the *Tp*-exposed THP-1 cells and PBS-treated control cells. In total, 1,477 mRNAs were significantly upregulated, and 1,137 mRNAs were significantly downregulated in the *Tp*-exposed THP-1 cells compared with control cells. The red dots indicate the mRNAs with upregulated expression, the blue dots indicate the mRNAs with downregulated expression, and the black dots indicate the mRNAs with no significant differences between groups. mRNAs with an expression fold change >2 and with an FDR-adjusted *P* < 0.05 were considered statistically significant. **(C)** GO analysis evaluated the pathways enriched by the top 20 dysregulated mRNAs.

To further elucidate the effects of *Tp* on monocytes, *Tp* (MOI = 10) was co-cultured with THP-1 cells for 12 h and transcriptome profiling by RNA-Seq was conducted. In total, 2,614 mRNAs were differentially expressed between the monocytes of three *Tp*-exposed monocyte groups and three PBS-treated controls, and those with a fold change greater than two were considered over-expressed or under-expressed (**Figure 2A**). After applying the criteria, specifically, 1,477 mRNAs were significantly upregulated, whereas 1,137 mRNAs were significantly downregulated in the *Tp*-exposed monocytes compared to PBS-treated monocytes (**Figure 2B**). There were 47 differentially expressed cytokines, of which three were downregulated, and 44 were upregulated. CXCL1, IL-1β, and TNF-α were the differentially regulated in *Tp* infected monocytes among the top 50 differential genes. In gene ontology (GO) analysis, which evaluates the enrichment of dysregulated mRNAs in cellular components, biological processes, and molecular functions, the most significant associations were observed with the following terms: immune system process, cell migration, and cell motility (**Figure 2C**).

### *Tp* Changes the Function of Monocytes THP-1

#### *Tp* Promoted Immune System Process Changes in Monocytes THP-1

According to the GO analysis results of transcriptome profiling, the differentially expressed genes in *Tp*-exposed THP-1 cells were mostly involved in immune system processes. Therefore, the changes in immune function in THP-1 cells caused by *Tp* were analyzed.

#### *Tp* Induced the Expression of IL-1β and TNF-α in THP-1 Cells

**Figure 3:**
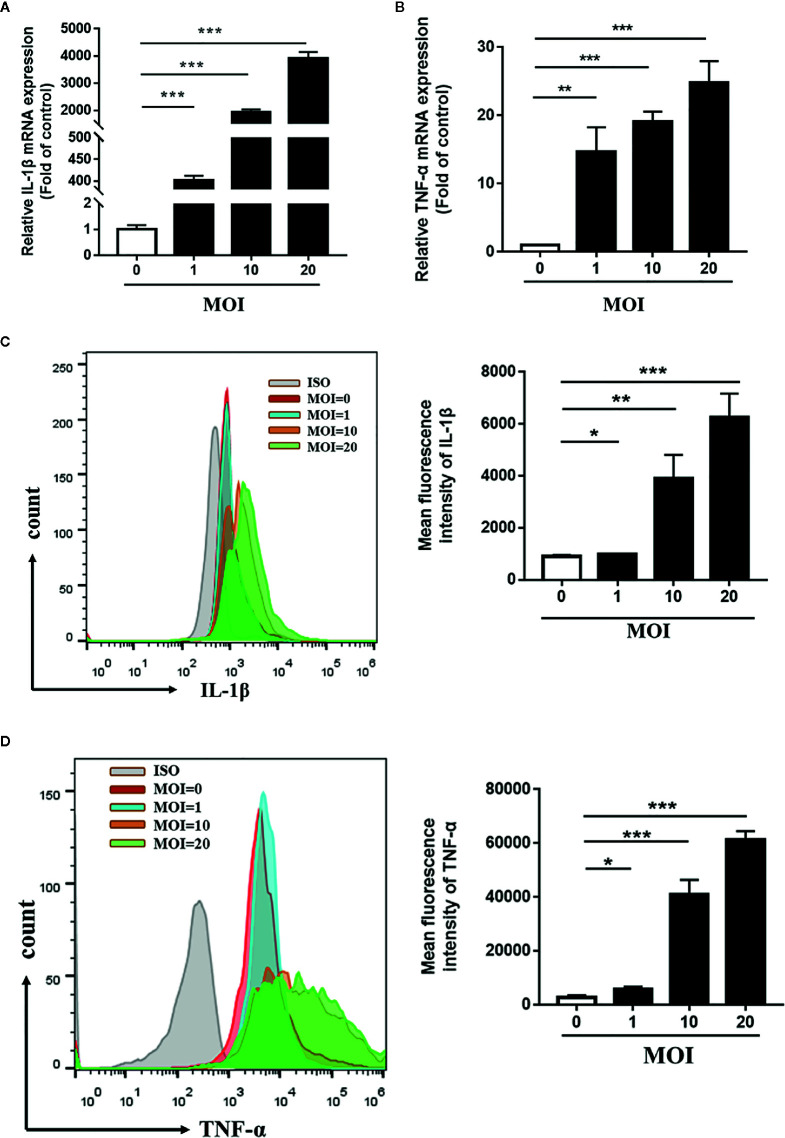
*Tp-*induced expression of IL-1β and TNF-α in THP-1 cells. THP-1 cells were incubated with *Tp* at different MOIs for 12 h. The mRNA expression of IL-1β and TNF-α was evaluated by RT-PCR **(A, B)**. The protein expression of IL-1β and TNF-α was evaluated by flow cytometry **(C, D)**. (*P < 0.05; **P < 0.01; ***P < 0.001). MOI, multiplicity of infection.

To verify the effects of *Tp* on monocyte function, we firstly detected the expression levels of IL-1β mRNA and TNF-α mRNA in THP-1 cells incubated with *Tp* at different MOIs. As shown in **Figures 3A, B**, the expression of IL-1β mRNA in *Tp*-treated monocytes at different MOIs (*Tp*: cells 1:1, 10:1 and 20:1) was significantly increased by 401.8 ± 6.01, 1,929 ± 60.47, and 3,909 ± 137.5 times compared to the control group, respectively. Meanwhile, the expression of TNF-α mRNA in monocytes increased significantly compared with the control group by 14.67 ± 2.07, 19.09 ± 0.85, and 24.77 ± 1.83 times (*P* < 0.001), respectively. Next, the expression levels of IL-1β protein and TNF-α protein were detected by flow cytometry. As shown in the **Figures 3C, D**, the average fluorescence intensities of IL-1β and TNF-α increased with increasing MOIs of *Tp*. This demonstrated a dose-dependent effect (*P* < 0.05).

## mTOR Was Essential for the Expression of IL-1β on *Tp*-Treated THP-1 Cells

**Figure 4:**
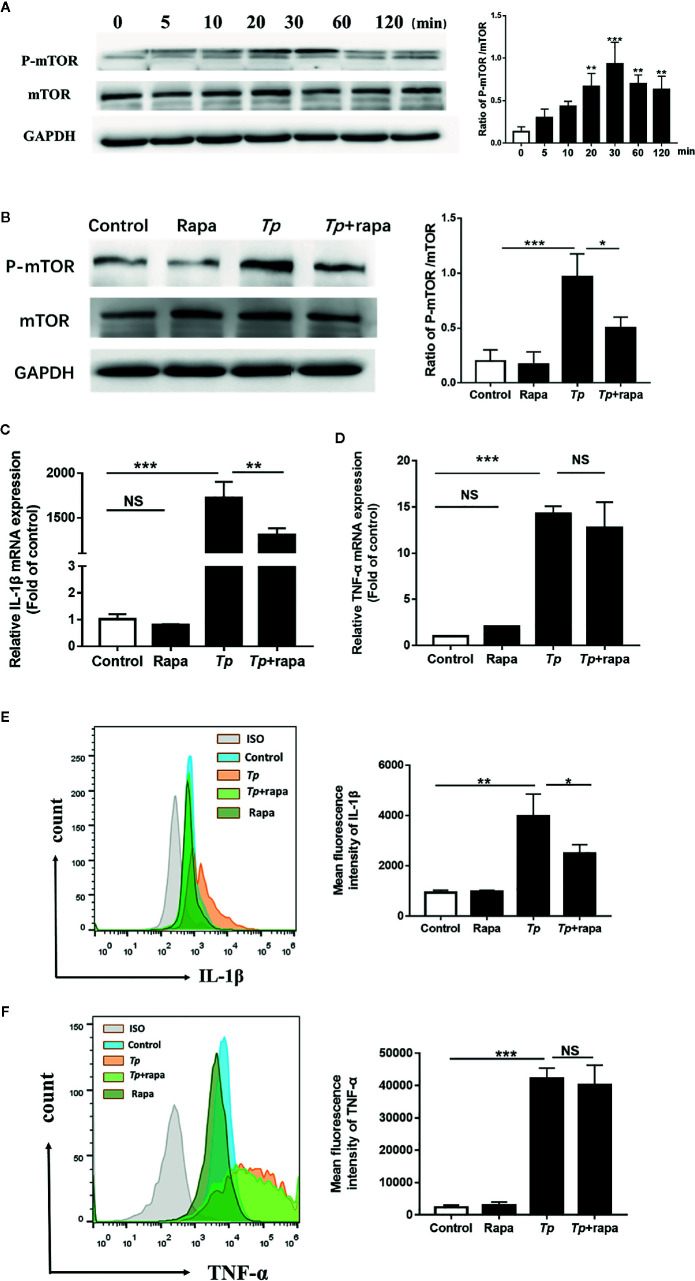
mTOR, but not autophagy, was essential for the expression of IL-1β induced by *Tp*. **(A)** THP-1 cells were incubated with *Tp* at an MOI of 10 for different amounts of time, and the levels of phosphorylated and total mTOR protein were detected by Western blotting. **(B)** THP-1 cells were pre-treated with Rapa (50 nM) for 30 min and then incubated with *Tp* at an MOI of 10 for 30 min. The levels of phosphorylated and total mTOR protein were detected by Western blotting. **(C, D)** THP-1 cells were pre-treated with Rapa (50 nM) for 30 min and then incubated with *Tp* at an MOI of 10 for 12 h. The levels of IL-1β and TNF-α were evaluated by RT-PCR. **(E, F)** The protein expression of IL-1β and TNF-α was detected by flow cytometry. (*P < 0.05; **P < 0.01; ***P < 0.001).

To explore the mechanism of *Tp*’s effect on monocyte function, we first evaluated the role of mTOR on monocyte function with *Tp*-treated cells by western blotting. The phosphorylation of mTOR (P-mTOR), a well-defined indicator of mTOR protein activation, was stimulated on monocytes by *Tp* in a time-dependent manner, and the concentration peak appeared at 30 min and then declined over time (**Figure 4A**). Next, THP-1 cells were pre-treated with Rapa (mTOR inhibitor), which inhibited the phosphorylation of mTOR (**Figure 4B**), and then attenuated the expression of IL-1β mRNA and IL-1β protein induced by *Tp* (**Figures 4C, E**). The mRNA expression and protein expression of TNF-α were also downregulated, but the differences were not significant (**Figures 4D, F**).

Autophagy is closely related to the secretion of inflammatory cytokines (Cadwell, 2016). In the present study, the autophagy level of *Tp*-exposed monocytes was significantly increased compared to that in the control group (**Supplementary Figure 1A**). However, when the autophagy was suppressed by pre-treatment with 3-MA (**Supplementary Figure 1B**), IL-1β and TNF-α expression showed no significant changes (**Supplementary Figures 1C–F**).

## Effect of *TP* on Phagocytosis of Monocytes THP-1

**Figure 5:**
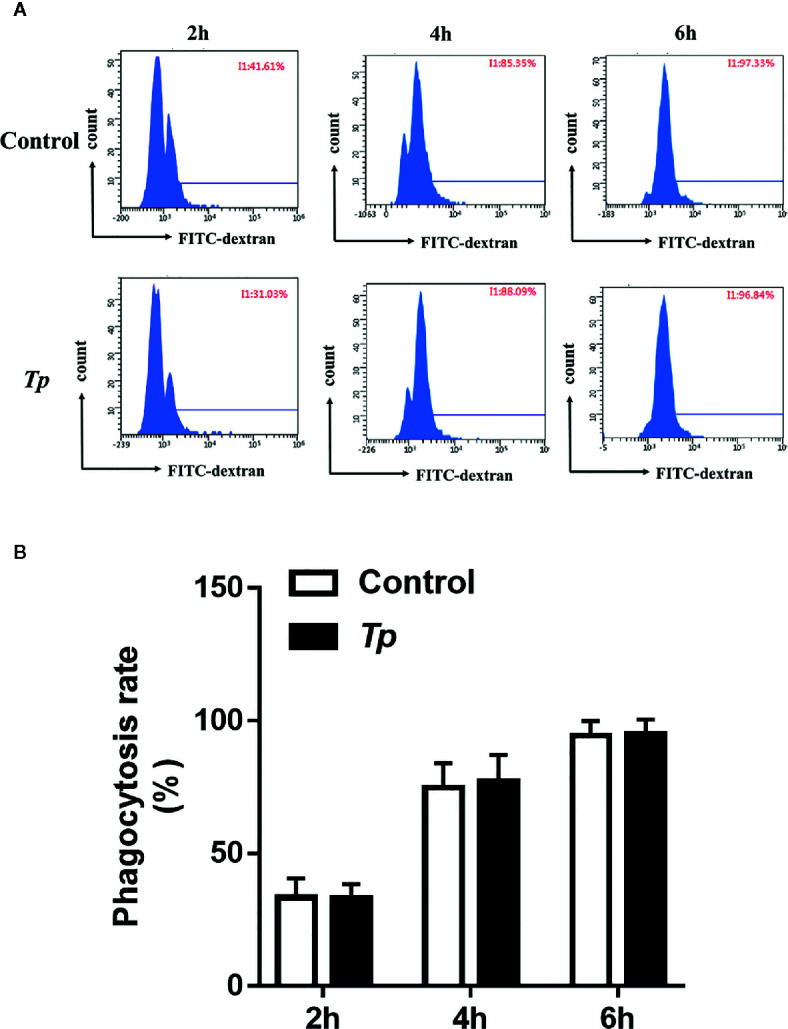
*Tp* had no effect on the phagocytosis of monocytes. **(A)** THP-1 cells were incubated with *Tp* at an MOI of 10 for 12 h, and then, the phagocytosis ability of monocytes was tested by flow cytometry. **(B)** The data in the bar graphs are the means ± SEMs of three separate experiments.

The phagocytosis of innate immune cells is essential for the clearance of pathogens invading the body. We used fluorescent microspheres to detect the phagocytic capacity of monocytes after *Tp* stimulation. After THP-1 cells were incubated with FITC-dextran for 2, 4, and 6 h; the phagocytic rates of the monocytes in the experimental group and the control group showed no significant differences (*P* > 0.05, **Figures 5A, B** and **Supplementary Figure 2**).

## *Tp* Promoted Monocyte THP-1 Migration

**Figure 6:**
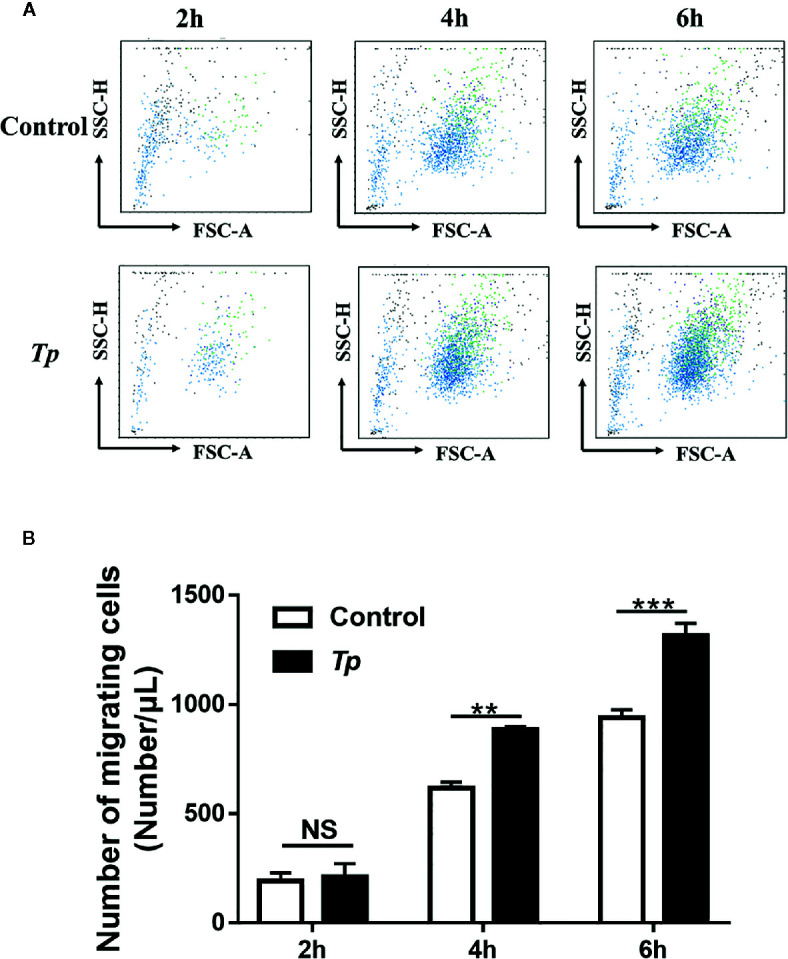
*Tp* promoted monocyte migration. **(A)** THP-1 cells were incubated with *Tp* at an MOI of 10 for 12 h. Then, the number of migrated cells was detected by using flow cytometry at different time points (2, 4, and 6 h). **(B)** The data in the bar graphs are the means ± SEMs of three separate experiments. (**P < 0.01; ***P < 0.001).

According to the GO analysis results of transcriptome profiling, the differentially expressed genes in *Tp*-exposed THP-1 cells were enriched in the primary function of cell migration. Therefore, we analyzed changes in *Tp*-induced THP-1 cell migration. To examine the effects of *Tp* on monocyte migration ability, we added RPMI 1640 medium with 10% FBS to the lower chambers of a Transwell system containing monocytes pre-exposed to *Tp* in the upper chambers. The number of cells pre-exposed to *Tp* that migrated to the lower chambers at the 4 and 6 h was significantly higher than that of the cells not pre-exposed to *Tp* that migrated to the lower chambers. There were no significant differences in the number of cells during the 2 h migration. The results are shown in **Figures 6A, B**.

## *Tp* Altered Monocyte THP-1 Migration *via* mTOR

**Figure 7:**
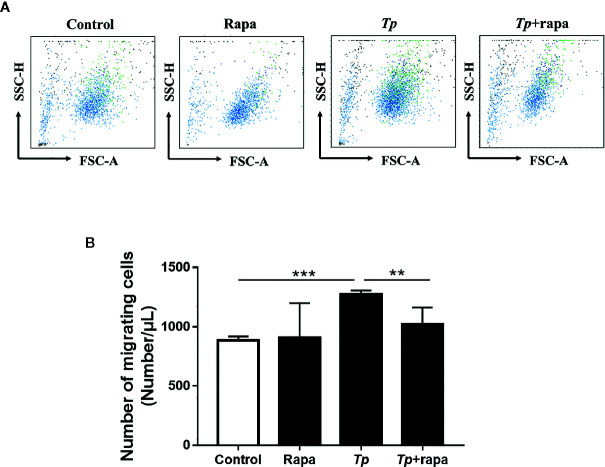
*Tp* altered monocyte migration *via* mTOR. **(A)** THP-1 cells were pre-treated with Rapa (50 nM) for 30 min and then incubated with *Tp* at an MOI of 10 for 12 h. Then, the number of cells that migrated to the lower chamber at 6 h was detected by using flow cytometry. **(B)** The data in the bar graphs are the means ± SEMs of three separate experiments. (**P < 0.01; ***P < 0.001).

After Rapa pre-treatment, THP-1 cells were cocultured with *Tp* for 12 h. The migrated monocytes in the lower chamber were collected at 6 h and counted by flow cytometry. Compared with that in the inhibitor-free group, the number of cells migrating to the lower chamber in the Rapa pre-treatment group was significantly reduced (*P* < 0.01, **Figures 7A, B**).

## Discussion

Abnormalities of monocyte subsets have been reported in many studies and was associated with a wide range of diseases and related to disease prognosis (Hirose et al., 2019; Nabatanzi et al., 2019). Our study found that syphilis patients demonstrate changes in monocyte subpopulations, of which intermediate monocytes were significantly increased. In addition, *Tp* could increase the expression of CD14 and CD16 in a human monocyte cell line (THP-1) *in vitro* and promote the transformation of monocytes into intermediate monocytes (CD14^++^CD16^+^ monocytes). Because intermediate monocytes controlled the differentiation of Treg subsets in HIV-1 coinfections (Guo et al., 2019), we speculated that the increase in intermediate monocytes in syphilis patients may be important for the differentiation of T lymphocyte subsets and the immune evasion of *Tp*. Studies have also found a significant increase in Treg cells in syphilis patients with sero-resistance (Zhao et al., 2016) and abnormal T lymphocyte subsets in neurosyphilis (Liu et al., 2019). In addition, upon viral infection, non-classical monocytes exhibited strong pro-inflammatory properties that skewed the immune response towards a Th2 profile (Kang et al., 2020). In agreement with our findings, the monocyte subpopulation in patients with *brucellosis* was also abnormal; what was different from our study was that the number of CD14^++^CD16^−^ monocytes was significantly higher in patients with brucellosis than the numbers of other monocyte subsets. This suggests that each monocyte subset plays divergent roles in different diseases, as well as differing in the ability to secrete cytokines.

Intermediate monocytes (CD14^++^CD16^+^ monocytes), compared with the classical monocyte subsets (CD14^++^CD16^−^ monocytes), showed a higher phagocytosis rate and secreted higher levels of IL-1β and TNF-α (Andrade et al., 2014). This study showed that *Tp* promoted IL-1β and TNF-α expression in monocytes THP-1, which was in agreement with a previous study (Babolin et al., 2011). IL-1β and TNF-α are pro-inflammatory cytokines that can cause inflammatory responses and tissue destruction. Through these inflammatory factors, immune cells such as neutrophils, macrophages, T lymphocytes, and B lymphocytes can be recruited, which leads to the inflammation of the skin and blood vessels (Gao et al., 2019). Inflammation in tissues is accompanied by the continued presence of *Tp* to maintain a strong immune response (Hawley et al., 2017). A study found that the levels of IL-1β, IL-6, and TNF-α were elevated in serum collected from syphilis patients (Peng et al., 2019). Although studies have shown the expression of *Tp*-induced pro-inflammatory cytokines (Zhu et al., 2017; Lin et al., 2018a; Lin et al., 2018b), very little is known about the *Tp*-mediated intracellular signaling pathway activation that leads to cytokine expression in monocytes.

To uncover the potential roles of monocytes in the occurrence of syphilis, a GO analysis was performed with the differentially expressed mRNAs in the *Tp*-exposed monocytes THP-1 and control cells. The differentially expressed mRNAs were mainly involved in biological processes. Among them, the most significant associations were observed with the following terms: immune system process, immune response, cell migration, response to cytokine, and cell activation, and these results are consistent with our results that *Tp* mainly affects monocyte migration and secretion of IL-1β and TNF-α; however, no significant effect on the phagocytic function of monocytes was observed. Radolf et al. (Moore et al., 2007) found that *Tp* had the ability to avoid detection and uptake by virtue of its denuded outer membrane. Cruz et al. (2012) observed that while human syphilitic sera promoted uptake of *Tp* in conjunction with monocyte activation, most *Tp* bacteria were not internalized. These results indicate that *Tp* does not affect the phagocytic system of monocytes, thus achieving the purpose of being able to escape immune clearance.

A subsequent KEGG pathway analysis also found the abnormal mTOR and autophagy signaling pathways in the *Tp*-induced monocytes. Mammalian target of rapamycin (mTOR) is a central regulator of growth and host immunity cells. Our previous research showed that the activation of the mTOR pathway in the pathological process of *Tp* infection caused the differentiation of macrophages (Zeng et al., 2016; Lin et al., 2018a). In this study, we further found that mTOR activated by *Tp* was involved in IL-1β secretion but not in TNF-α expression. It proved that *Tp* regulated IL-1β secretion through the mTOR pathway. In addition to the mTOR signaling pathway, recent studies have found that autophagy is closely related to inflammation (Matsuzawa et al., 2012; Bah and Vergne, 2017; Wang XL et al., 2017), which represents a major adaptive response for maintaining cellular and tissue homeostasis. Our research found that *Tp* also significantly increased the level of autophagy in monocytes THP-1, but autophagy had no effect on the secretion of cytokines IL-1β and TNF-α stimulated by *Tp*. Autophagy regulates cytokine secretion depending on the cellular context. Pun et al. (2015) found that autophagy decreased TNF-α expression in primary macrophages. However, in human peripheral blood mononuclear cells stimulated with LPS, 3-MA decreased the transcription of TNF-α while upregulated the transcription of IL-1β (Crisan et al., 2011). The effect of autophagy on the inflammatory status was different in cells and pathogens. Based on our current results, *Tp*-induced autophagy had no effect on the inflammatory response. The role of *Tp*-induced autophagy needs to be further studied.

Inflammatory responses following exposure to a stimulator are highly dependent on the migration of monocytes. We found that *Tp* increased the migration ability of monocytes and promoted the expression of monocyte migration-related genes through GO analysis. Enhanced monocyte migration exacerbates the formation of tissue inflammation injury. A large number of monocytes, T lymphocytes, and other immune cells accumulate in syphilis skin lesions (Cruz et al., 2012). *Tp* promoted the expression of intercellular adhesion molecules in endothelial cells, the overexpression of matrix metalloproteinases, and the recruitment of monocytes to endothelial cells (Gao et al., 2019; Zang et al., 2019). However, Zhang et al. (Hu et al., 2020) found that *Tp* stimulated macrophages to produce exosomes containing miR-146a-5p, which reduced the expression of adhesion-reducing molecules in endothelial cells, as well as endothelial migration of monocytes. The effects of *Tp* on the migration of monocytes were varied. Our experiments started with *Tp*-stimulated monocytes and revealed a novel fact in syphilis pathophysiology. Our results showed that mTOR activated by *Tp* was involved in monocyte migration. After adding rapamycin, an inhibitor of mTOR protein, the *Tp*-induced monocyte migration ability was significantly inhibited.

In this study, we investigated the effect of *Tp* on peripheral monocyte subsets and functions *in vitro*. *Tp* induces the transition of monocytes into intermediate subpopulations (CD14^++^CD16^+^ monocytes), promotes monocyte secretion of IL-1β, and increases monocyte migration capacity through the mTOR pathway. These data may be useful for understanding the pathogen–host relationship and the pathogenesis of syphilis. Further studies are required to understand the role of monocytes in *Tp*-infected animals. In addition, the analysis of monocyte subsets in syphilis at different clinical stages would better elucidate the pathogenesis of *Tp*. Meanwhile, *Tp* might also affect other pathways in THP-1 cells, such as pathways involving phosphatases, and further studies are needed for related mechanisms.

## Data Availability Statement

The original contributions presented in the study are included in the article/**Supplementary Materials**. Further inquiries can be directed to the corresponding author.

## Ethics Statement

The studies involving human participants were reviewed and approved by the ethics committee of Zhongshan Hospital, Xiamen University. The patients/participants provided their written informed consent to participate in this study.

## Author Contributions

W-NL and L-LL conceived and designed the experiments. W-NL performed the experiments. W-NL, K-XW, and X-HS analyzed the data. X-YJ, Y-ZX, X-LH, YL, L-RL, and M-LT contributed reagents, materials, and analysis tools. W-NL, YL, and L-LL wrote the paper. All authors contributed to the article and approved the submitted version.

## Funding

This work was supported by the National Natural Science Foundation of China (grant numbers 81971147, 81771312, 81471231, 81101324, 81672094, 81871729, 81972028). The funders played no role in the study design, data collection, or analyses, the decision to publish, or manuscript preparation.

## Conflict of Interest

The authors declare that the research was conducted in the absence of any commercial or financial relationships that could be construed as a potential conflict of interest.

## Supplementary Material

Supplementary Figure 1 Autophagy was not involved in the expression of IL-1β induced by *Tp*. **(A, B)** THP-1 cells were incubated with *Tp* at an MOI of 10 for 12 h with or without pre-treatment with 3-MA (1 mM) for 30 min, and the levels of LC3 were detected by western blotting. **(C, D)** THP-1 cells were pre-treated with 3-MA (1 mM) for 30 min and then incubated with *Tp* at an MOI of 10 for 12 h. The levels of IL-1β and TNF-α were evaluated by RT-PCR and **(E, F)**. The protein expression of IL-1β and TNF-α was detected by flow cytometry.

Supplementary Figure 2
*Tp* had no effect on the phagocytosis of monocytes. THP-1 cells were incubated with *Tp* at an MOI of 10 for 12 h, and then, the phagocytosis ability of monocytes was tested by flow cytometry showed in one histogram from different time points.

## Abbreviations

FSC, forward scatter; SSC, side scatter; HC, healthy controls; SP, syphilis patients; *Tp, Treponema pallidum*; HIV, human immunodeficiency virus; HBV, hepatitis B virus; HCV, hepatitis C virus; RPR, rapid plasma regain; TPPA, treponema pallidum gelatin; agglutination; ANOVA, one-way analysis of variance; MOI, multiplicity of infection; Rapa, rapamycin; mTOR, mammalian target of rapamycin.
