# Supplementary figures and images for: Treponema pallidum Dysregulates Monocytes and Promotes the Expression of IL-1β and Migration in Monocytes Through the mTOR Signaling Pathway
Source: Front Cell Infect Microbiol. 2020 Nov 13;10:592864. doi: 10.3389/fcimb.2020.592864 (PMC7691244; doi:10.3389/fcimb.2020.592864)

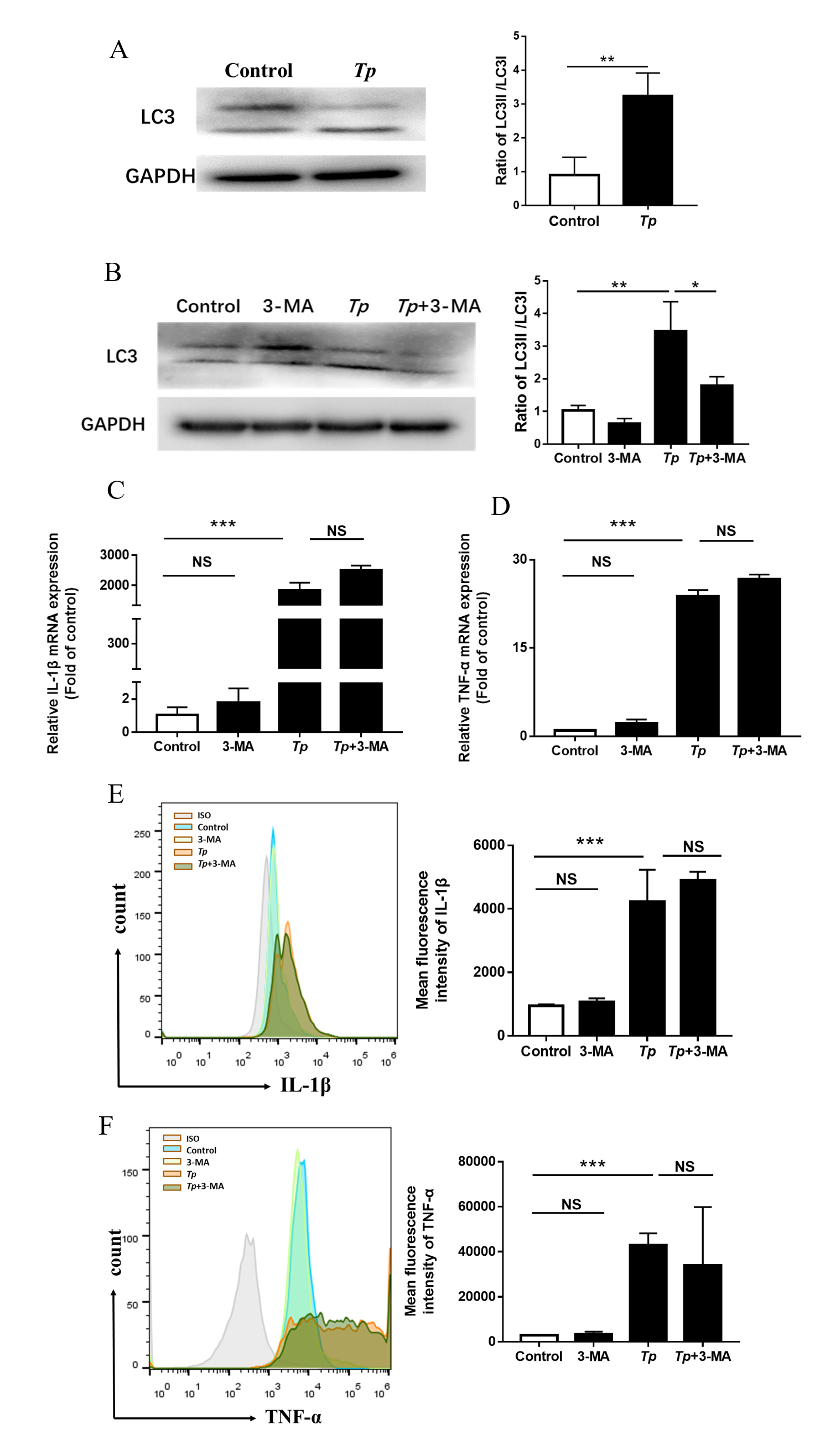

Supplement: Supplementary Figure 1 — Autophagy was not involved in the expression of IL-1β induced by Tp. (A, B) THP-1 cells were incubated with Tp at an MOI of 10 for 12 h with or without pre-treatment with 3-MA (1 mM) for 30 min, and the levels of LC3 were detected by western blotting. (C, D) THP-1 cells were pre-treated with 3-MA (1 mM) for 30 min and then incubated with Tp at an MOI of 10 for 12 h. The levels of IL-1β and TNF-α were evaluated by RT-PCR and (E, F). The protein expression of IL-1β and TNF-α was detected by flow cytometry. [file Image_1.tif]

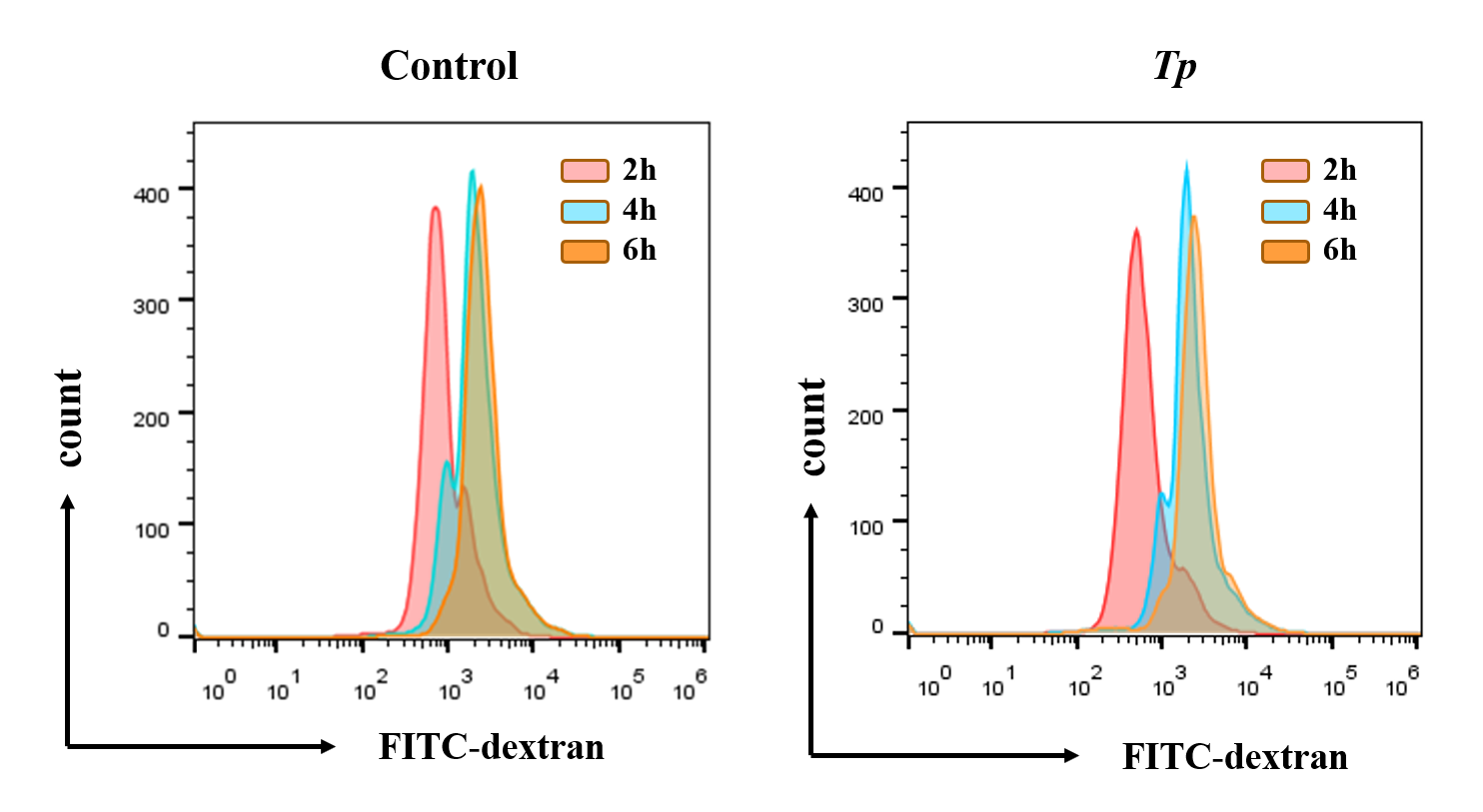

Supplement: Supplementary Figure 2 — Tp had no effect on the phagocytosis of monocytes. THP-1 cells were incubated with Tp at an MOI of 10 for 12 h, and then, the phagocytosis ability of monocytes was tested by flow cytometry showed in one histogram from different time points. [file Image_2.tif]
