# Supplementary material for: Treponema pallidum Dysregulates Monocytes and Promotes the Expression of IL-1β and Migration in Monocytes Through the mTOR Signaling Pathway
Source: Front Cell Infect Microbiol. 2020 Nov 13;10:592864. doi: 10.3389/fcimb.2020.592864 (PMC7691244; doi:10.3389/fcimb.2020.592864)
Supplement: Supplementary file 3 [file Table_1.docx]

**Table 1.** Primer sequences of the target gene

| **Gene** | **Oligonucleotide primer sequences (5′-3′)** |
| --- | --- |
| TNF-α | Forward ATGAGCACTGAAAGCATGAT |
|  | Reverse GGGCTGATTAGAGAGAGGTC |
| IL-1β | Forward GATGGCTTATTACAGTGGC |
|  | Reverse CCTTGCTGTAGTGGTGGT |
| GAPDH | Forward GAAGGTGAAGGTCGGAGTC |
|  | Reverse GAAGATGGTGATGGGATTTC |

**Table 2** Basic information of the participants

|  | **Syphilis patients** | **Health controls** | ***P*** |
| --- | --- | --- | --- |
| n | 55 | 55 | - |
| Male/female | 32/23 | 23/32 | *P*>0.05 |
| Age (median, interquartile range) | 56, 18 | 52, 13 | *P*>0.05 |
| Positive serum RPR | 100% | 0% | - |
| Positive serum TPPA | 100% | 0% | - |
